# Determinants of diagnostic latency in Chinese people with Parkinson’s disease

**DOI:** 10.1186/s12883-019-1323-5

**Published:** 2019-06-11

**Authors:** Ying Wan, Yingying Zhu, Yi Luo, Xun Han, Yongsheng Li, Jing Gan, Na Wu, Anmu Xie, Zhenguo Liu

**Affiliations:** 1 Department of Neurology, Xinhua Hospital, Affiliated to Shanghai JiaoTong University, School of Medicine, 1665 Kongjiang Road, Shanghai, 200092 China; 2 Department of Neurology, affiliated Hospital of Medical College Qingdao University, Qingdao, 266000 Shandong Province China

**Keywords:** Parkinson’s disease, Diagnostic latency, Determinants, Non-motor symptoms, Strategies

## Abstract

**Background:**

Clinical diagnosis of Parkinson’s disease (PD) has always lagged behind clinical symptoms. The diagnostic latency might be influenced by many factors. The diagnostic latency of Chinese people with PD has been unknown. Here we designed this cross-sectional study with the purpose to identify the diagnostic latency and its determinants in Chinese people with PD.

**Methods:**

One hundred and thirty-one newly diagnosed people with PD were recruited into this study. Demographic and clinical characteristics as well as a detailed clinical history were collected. Motor and non-motor symptoms (NMSs) severity were assessed with appropriate assessment scales. Medical professional types in the first medical consultations were also recorded. According to the initially presenting motor phenotypes, patients would be divided into the groups of rest tremor, limb rigidity, movement slowness and walking problems. The investigated variables would be compared among the four groups.

**Results:**

The PD diagnostic latency in China was around 15 months. It closely correlated to the severity of motor symptoms, anxiety and depression as well as the number of NMSs. The diagnostic latency significantly varied among the groups of different motor phenotypes of onset. Finally, initially presenting with limb rigidity, having more NMSs, motor symptoms at a more serious degree and the initial medical consultations with physicians or specialists of non-neurology were considered as determinants of a longer diagnostic latency of PD.

**Conclusions:**

Patients presenting with minor motor symptoms and disturbing NMSs as well as physicians’ unfamiliarity with PD symptomology were determinants of the diagnostic delay of PD. Health education in community and improvement of the referral system might be proper strategies to shorten the diagnostic latency of PD.

## Background

Parkinson’s disease (PD) is well known for the cardinal motor symptoms including rest tremor, limb rigidity, global bradykinesia, postural and gait disturbance [1]. In addition, people with PD suffer from a series of non-motor symptoms (NMSs). Some of NMSs occur years before motor symptoms and have been reported as prodromal signs of PD. [2, 3] All these motor and NMSs could lead to a great burden on the quality of life of both patients and their caregivers [4, 5]. So far, the dopamine-replacement therapy has been considered as a quite effective strategy in alleviating clinical symptoms. A timely diagnosis and treatment would be helpful for patients to improve their daily living abilities and the quality of life. According to the previous studies, the time span from the motor symptom occurrence to clinical diagnosis of PD ranged from months to years [6, 7]. The PD diagnostic latency was reported to be influenced by the demographic and clinical characteristics [7]. However, the relationship between the diagnostic latency and the NMSs has not been studied. Currently, the diagnostic latency in China has not been reported. Interestingly, the medical service in China has its own feature as it provides a patient with an opportunity of a clinical consultation with a specialist without the referral from a physician. Whether this feature of the medical system in China might shorten the time span for PD diagnosis has been unknown. Hence, we designed a cross-sectional investigation in two public hospitals to identify the diagnostic latency of PD and its determinants.

## Methods

### Study population

Between June 2013 and May 2016, 131 patients were consecutively recruited into the study. The patients were newly diagnosed with PD in the Movement Disorders Clinics at the Department of Neurology of Xinhua Hospital affiliated to Shanghai Jiaotong University School of Medicine (Shanghai) and affiliated Hospital of Qingdao University (Shan Dong Province). All participants were diagnosed with idiopathic PD according to the UK PD brain bank criteria [8]. Patients who were unable to complete the questionnaires would be excluded from the study. Since we investigated the diagnostic latency in two cities, patients would also be excluded from the study if they were not local residents.

### Study design

A standardized questionnaire was designed for this study. The questionnaire was composed of three sections that covered demographic characteristics, a detailed clinical history and the information on the medical professionals during the time from the first medical consultations to PD diagnosis. Each section contained a series of questions in the combination of options and blanks. The participant and his or her caregiver were requested to finish the questionnaire together. The caregivers in our study referred to the people who had lived with the participants, such as the spouses or offspring. For the participants at the solo living status, the caregivers referred to the people who had provided long-term care services for the participants before the recruitment, such as the siblings or nurses. Demographic characteristics include age, sex, education background (educated or not), career (physical or mental working), smoking, family history of PD, dwelling state (solo living or cohabiting), working status (retired or not) and whether the dominant side was affected when the motor symptom occurred. The clinical history included the initially presenting motor phenotype, the date of the motor symptom onset (when the patient or the caregiver considered the motor symptom as being abnormal), and the dates of the first medical consultation and PD diagnosis. All the dates were recorded by month. Based on the previous study [9], the initially presenting motor phenotypes, defined as the predominant complaints that drove a patient to seek medical help, would be classified into four types in our study: rest tremor, limb stiffness, walking problems and movement slowness. The comorbidities were also noted and classified into six categories according to the methods in Marrie’ study [10].

Clinical characteristics including the severity of motor and NMSs would be assessed in the 131 people with PD by employing appropriate scales. The Unified Parkinson’s Disease Related Scale motor part (UPDRS-III) and Hoehn & Yahr (H&Y) staging were used to assess the severity of motor symptoms. Non-motor symptoms questionnaire (NMSquest) was used to assess the numbers of non-motor symptoms (NMS). Mini-mental State Exam (MMSE) was used to assess the severity of cognitive impairment; Hamilton Anxiety Scale (HAMA) and Hamilton Depression Scale (HAMD) were used to assess the severity of anxiety and depression. The investigation was conducted through face-to-face inquiries. All assessments were performed by well-trained movement disorder neurologists. Patients would be divided into four groups according to the initially presenting motor phenotypes. The investigated variables would be compared among the four groups.

### Statistics

Descriptive statistics were used as required. Since all parameters did not follow a normal distribution, non-parametric methods were used in the data analysis. Continuous data were presented as medians and quarters and categorical variables were presented as numbers and percentages. Kruskal-Wallis test was used for multiple comparisons of continuous variables among different groups (*n* ≥ 3). The post hoc test was evaluated with the Nemenyi test. Spearman rank correlation coefficient was used to calculate the correlation between the continuous variables. Kaplan-Meier curves and the log-rank test were used to calculate the correlation between initially presenting motor phenotypes and the diagnostic latency of PD. Cox proportional hazards models were used to screen the key factors of the diagnostic delay of PD in the univariate and multivariate analyses. Statistical significance was set at *p* < 0.05, with a two-tailed approach. Statistical computations were performed by SPSS.

## Results

### Characteristics of the population

The demographic and clinical characteristics of the four groups were showed in Tables 1 and 2. Rest tremor was the most common motor phenotype of onset whereas walking problems was the least common motor phenotype of onset. Patients in the group of walking problems were at older onset age than patients in the groups of rest tremor and limb rigidity (*p* < 0.01). Patients in the group of movement slowness were also at older onset age than patients in the group of limb rigidity (*p* < 0.01). The distribution of the other demographic characteristics remained the same among the four groups (Table 1). The severity of the motor and non-motor symptoms varied significantly among the four groups. The results of the comparisons among the four groups were showed in details in Table 2. Patients in the group of rest tremor performed best in the evaluations of the UPDRS-III, NMSquest, HAMA and HAMD (p < 0.01). By contrast, patients in the group of walking problems performed worst in the evaluations of UPDRS-III, HAMA, HAMD and MMSE (*p* < 0.05).

**Table 1:** Characteristics classified by the initially presenting motor phenotype

	Rest tremor (*n* = 65) A	Limb rigidity (*n* = 32) B	Walking problems (*n* = 15) C	Movement slowness (*n* = 19) D	P	Post hoc significance
Demographic features						
Male^#, a^, (n/%)	27 (41.5%)	22 (68.8%)	6 (40.6%)	11 (57.9%)	0.059	/
Onset age^※,b^	64 (59,72)	60 (55,68)	72 (66,74)	68 (59,73)	**< 0.001**	A < C**, B < C**, B < D**
Educated^#, a^, (n/%)	53 (81.5%)	32 (100%)	12 (80.0%)	17 (89.5%)	0.064	/
Retired^#, a^, (n/%)	45 (69.2%)	17 (53.1%)	13 (86.7%)	13 (68.4%)	0.134	/
Cohabiting^#, a^, (n/%)	60 (92.3%)	28 (87.5%)	13 (86.7%)	18 (94.7%)	0.739	/
Smoking^#, a^, (n/%)	23 (35.4%)	14 (43.8%)	3 (30.0%)	6 (35.1%)	0.447	/
Dominant side affected^#, a^, (n/%)	35 (53.8%)	17 (53.1%)	10 (66.7%)	9 (47.4%)	0.726	/
Mental working^#, a^, (n/%)	29 (44.6%)	15 (46.9%)	8 (53.3%)	9 (47.4%)	0.944	/
Family history^#, a^, (n/%)	4 (6.2%)	4 (12.5%)	3 (20.0%)	1 (5.3%)	0.308	/

Bold values indicated statistically significant values

^#^results were presented as number (percentage); ^a^analyzed by χ2 test; ** = *p* < 0.01

^※^results were presented as presented as medians (quarters); ^b^analyzed by by Nemenyi test

**Table 2:** Clinical characteristics and diagnostic latency classified by the initially presenting motor phenotype

	Rest tremor (*n* = 65) A	Limb rigidity (*n* = 32) B	Walking problems (*n* = 15) C	Movement slowness (*n* = 19) D	P	Post hoc significance
UPDRS-III score^※,b^	8 (6,11)	21 (18,24)	36 (29,41)	27 (23,38)	**< 0.001**	A < B**, A < C**, A < D**, B < C**, B < D**, D < C*
H&Y grade^#, a^					**/**	/
(0~1]	42 (64.6%)	0	0	0		
(1~2]	23 (35.4%)	32 (100%)	9 (60.0%)	17 (89.5%)		
> 2	0	0	6 (40.0%)	2 (10.5%)		
NMSquest score^※,b^	3 (2,6)	7 (4,10)	8 (4,10)	9 (6,13)	**0.001**	A < B**, A < C**, A < D**, B < D**
HAMA score^※,b^	5 (2,8)	8 (5,11)	13 (8,15)	6 (4,10)	**0.002**	A < B**, A < C**, A < D*, B < C*, D < C**
HAMD score^※,b^	5 (2,10)	10 (6,12)	18 (12,21)	11 (5,18)	**< 0.001**	A < B*, A < C**, A < D**, B < C**, D < C**
MMSE score^※,b^	28 (26,30)	29 (26,30)	26 (25,28)	28 (27,30)	**< 0.001**	C < A**, C < B**, C < D**
Time A^※,b^ (months)	4 (2,6)	10 (5,12)	6 (5,10)	16 (11, 21)	**< 0.001**	A < B**, A < C**, A < D** B < D**, C < D**
Time B^※,b^ (months)	6 (4,7)	11 (9,18)	16 (12,18)	8 (6.5, 15)	**< 0.001**	A < B**, A < C**, A < D**, B < C*, D < B**, D < C**
Time C^※,b^ (months)	10 (7,12)	22 (17,27)	24 (20,27)	27 (23.5, 31)	**< 0.001**	A < B**, A < C**, A < D**,

^#^results were presented as percentage

^※^results were presented as presented as medians (quarters)

^a^analyzed by χ^2^ test

^b^analyzed by by Nemenyi test; * = *p* < 0.05; ** = *p* < 0.01; Bold values indicated statistically significant values; UPDRS = Unified Parkinson Disease

Rating Scale; H&Y grade = Hoehn &Yahr grade; NMSquest = Non-motor symptoms questionnaire; HAMA = Hamilton Anxiety

Scale; HAMD = Hamilton Depression Scale; MMSE: Mini mental State Exam; Time A = the time from motor symptom onset to patients’ initiation of medical consultations; Time B = the time from patients’ first medical consultations to PD diagnosis; Time C = the time from motor symptom onset to clinical diagnosis of Parkinson’s disease

### The diagnostic latency of PD

The median duration from the motor symptom onset to PD diagnosis was around 15 months (ranged from 4.0 to 42.5 months). The duration from the motor symptom onset to the initiation of medical consultations (Time A, the median duration = 6 months) was a bit shorter than the duration from the initiation of medical consultations to being diagnosed with PD (Time B, the median duration = 7.5 months). Further analysis showed that the duration from the motor symptom onset to PD diagnosis positively correlated with the scores of UPDRS -III, NMS quest, HAMA and HAMD (*p* < 0.001). However, it was not associated with the onset age or the MMSE score (Table 3).

**Table 3:** Correlation between functional impairment and the diagnostic latency

Variables	Time C
Onset age ^a^	0.088 (0.315)
UPDRS-III score ^a^	0.846(< 0.001)**
NMSquest score ^a^	0.615(< 0.001)**
HAMA score ^a^	0.321(< 0.001)**
HAMD score ^a^	0.361(< 0.001)**
MMSE score ^a^	−0.115 (0.195)

Results were presented as ‘rho (*p* value)’; ^a^analyzed by spearman correlation; ** = *p* < 0.01; Bold values indicated statistically significant values; UPDRS = Unified Parkinson Disease Rating Scale; NMSquest = Non-motor symptoms questionnaire; HAMA = Hamilton Anxiety Scale; HAMD = Hamilton Depression Scale;

### The distribution of medical professional types in the first medical consultations

**Fig. 1:**
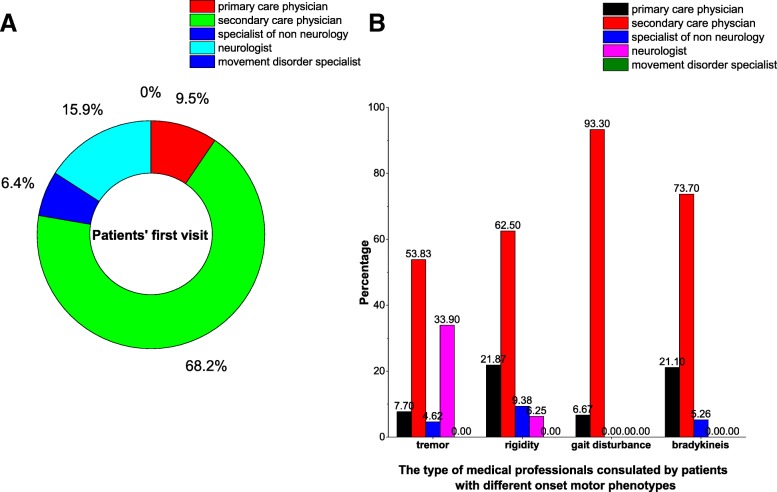
**a** the distribution of medical professionals that initially consulted by patients; **b** the distribution of medical professionals that initially consulted by patients among different motor phenotypes

The distribution of medical professional types in the first medical consultations was showed in Fig. 1a and b. Secondary care physicians accounted for the largest portion of the medial professional types in the first medial consultations (83/131, 63.36%). By contrast, movement disorder specialists accounted for zero in the first medical consultations (Fig. 1a). Interestingly, 18.32% (24/131) of people with PD started medical consultations with neurologists. Most of these patients initially presented with rest tremor (*n* = 22). However, no patients initially presenting with gait disturbance or movement slowness started medical consultations with neurologists (Fig. 1b).

### Initially presenting motor phenotype and the diagnostic latency of PD

**Fig. 2:**
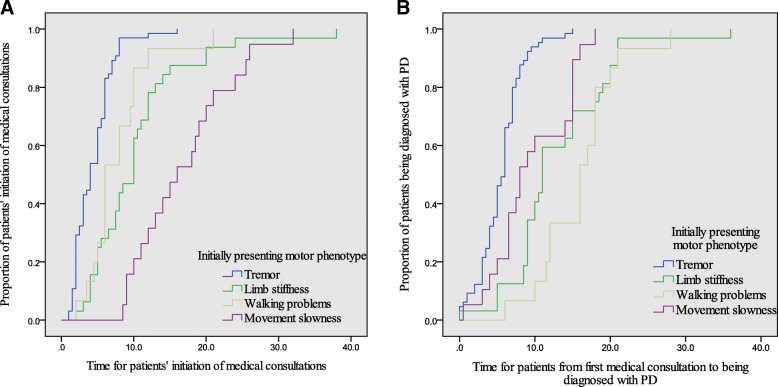
Kaplan-Meier curves showed **a** the time to patients’ initiation of medical consultations by the motor phenotype; **b** the time from the first medical consultation to PD diagnosis by the motor phenotype

Both the time A and the time B varied significantly among the four different motor phenotypes (Table 2). Here we analyzed the correlation between the four motor phenotypes and the two targeted time intervals with the Kaplan-Meier curves and the log-rank test. The risks of having a longer time interval in the two periods varied significantly among the four groups (*p* < 0.05). In terms of the time A, the group of movement slowness was at the highest risk of a delayed initiation of medical consultations whereas the group of rest tremor was at the lowest risk. The time A by the motor phenotype was displayed in Fig. 2a. In terms of the time B, both the groups of walking problems and limb stiffness were at the highest risks of a longer time interval in this period whereas the group of rest tremor was at the lowest risk. The time from the first medical consultations to being diagnosed with PD by the initially presenting motor phenotype was displayed in Fig. 2b.

### Determinants of a longer diagnostic latency of PD

The variables were separately included into a Cox proportional hazards model through a univariate analysis. The variables with p < 0.05 (including initially presenting motor phenotypes, the NMSquest score, the UPDRS-III score, the HAMA score, the HAMD score and the medical professional types in the first medical consultations) were all included into the multivariable Cox analysis. Finally, the factors of initially presenting motor phenotypes (limb rigidity versus tremor), a higher NMSquest score and a higher UPDRS-III score were considered as determinants of a longer diagnostic latency of PD in the model (Table 4).

**Table 4:** Determinants of the targeted time intervals in the multivariate COX model

Variables	HR	95%CI	p
Time C			
Initially presenting motor phenotype			
Limb stiffness vs rest tremor	0.168	0.081~0.347	< 0.001**
Walking problems vs rest tremor	0.717	0.223~2.309	0.578
Movement slowness vs rest tremor	0.419	0.151~1.167	0.096
NMSquest score	0.871	0.815~0.930	< 0.001**
UPDRS motor score	0.916	0.883~0.950	< 0.001**
HAMA score	0.965	0.901~1.034	0.312
HAMD score	1.049	0.993~1.107	0.087
The first medical consultation with physicians or specialists of non-neurology	0.346	0.198~0.606	< 0.001**

** = *p* < 0.01

HR = hazard ratio; CI = confidence interval; Bold values indicated statistically significant values; UPDRS = Unified Parkinson Disease Rating Scale; NMSquest = Non-motor symptoms questionnaire; HAMA = Hamilton Anxiety Scale; HAMD = Hamilton Depression Scale; Time A = the time from motor symptom onset to patients’ initiation of medical consultations; Time B = the time from patients’ first medical consultation to PD diagnosis; Time C = the time from motor symptom onset to clinical diagnosis of Parkinson’s disease

## Discussion

This was the first report of the diagnosis latency in Chinese people with PD. Our results found that patients in China spent around 15 months from motor symptom onset to PD diagnose, which was much shorter than Chilean and Mexican reports [11, 12]. However, it was still longer than the diagnostic latency (12 months) in Britain [7]. Actually, the diagnostic latency of PD varied widely among different studies. The longest diagnostic latency reported was more than 100 months [7]. A longer diagnostic latency always resulted in a more serious motor functional impairment. In our study, more than half of the newly diagnosed patients (68/131) were at H&Y grades of II and III, with a significant decline of the motor function and self-care abilities [11]. Our results indicated that proper strategies should be designed to reduce the diagnostic latency to ensure that Chinese people with PD could acquire the timely medical treatment, improving the quality of life.

According to our results, the diagnostic latency of PD was determined by the initially presenting motor phenotypes. The significant variance in the diagnostic latency of different initially presenting motor phenotypes might be attributed to the following two reasons: on one hand, the majority of PD population are aged people and the motor symptoms occur gradually and insidiously. Among the motor symptoms, rest tremor is easily detectable whereas the other motor symptoms including limb stiffness, walking problems and movement slowness are prone to be unnoticed and misinterpreted to aging by patients themselves. Therefore, patients initially presenting with rest tremor might tend to seek medical help, compared with patients initially presenting with the other motor symptoms. Here we suggested to spread the knowledge of PD and simple self-physical examinations in communities to help aged people to detect the body abnormality earlier, which might facilitate an earlier initiation of the medical consultations. On the other hand, the consulted medical professionals might have different familiarity with the four motor symptoms. We found that physicians were the majority of the medical professional types in the first medial consultations. Interestingly, patients in the groups of walking problems and limb rigidity were both at the highest risks of the referral delay. The results indicated that the symptoms of walking problems and limb rigidity might be less considered as an important feature of PD in physicians’ impression. Our results showed that the PD diagnostic latency would be delayed when patients initially consulted with physicians for their motor symptoms. Therefore, it would be helpful to improve physicians’ familiarity with PD’s symptomology to increase the effective referrals, shortening the time for PD diagnosis.

Our study investigated the relationship between the NMSs and the diagnostic latency of PD. The results indicated that patients with more NMSs experienced a longer diagnostic latency of PD. Actually, a lot of NMSs were identified to precede or accompany the occurrence of motor symptoms [13, 14]. The NMSs, especially at the early stage of PD, were more apparent and disturbing than motor symptoms for aged people and constituted the main complaints of People with PD in primary care institutes [15, 16]. The impact of NMSs on the quality of life was much greater than that of motor symptoms [17], leading to the ignorance of motor symptoms and delayed medical consultations for motor symptoms. Since a series of NMSs were identified to be prodromal signs of PD [18, 19], it would be more meaningful to conduct a long-term follow up of the aged people with such NMSs in communities in order to achieve an early diagnosis of PD and the disease modifying therapy [20, 21].

In addition to the factors discussed above, patients with higher UPDRS motor scores would be at a higher risk of being delayed for PD diagnosis. We thought the probability of incorrect referral delays would be significantly higher when physicians encountered patients with more complicated motor symptoms. It might furtherly lead to the diagnostic delay.

Although the medical system in China provided a patient with an opportunity of consultation with a neurologist or a movement disorder specialist without any referral, it did not effectively shorten the time from the first medical consultations to PD diagnosis. Physicians were still the majority of the medical professional types in the first medial consultations. PD diagnosis is a complicated process in which an intact knowledge of movement disorders should be used. It would be more reasonable for physicians to take the responsibility of providing the patients with a proper referral rather than making the diagnosis of PD. However, our current medical system has not clearly defined the responsibilities of different medical professional types as Europe [21, 22]. It might result in the increase of misdiagnosis rate and the diagnostic delay. Here we suggested to develop more effective strategies to improve the current medical system, reducing the diagnostic latency in PD.

Our studies had some limitations. The diagnostic latency would also be influenced by a lot of social and personal factors such as the convenience of medical service, the family income, the relationship between patients and their caregivers, which were not included into our study. Moreover, the participants in this retrospective study, seldom recalled the studied events to the exact date, which might decrease the accuracy of our results.

## Conclusions

The diagnostic latency of PD in China was determined by the factors of initially presenting motor phenotypes, the severity of motor symptoms, the numbers of accompanied NMSs as well as the medical professional type in the first medical consultations. Strengthening PD related health education and screens of Parkinson’s early motor and non-motor signs in communities and improving the medical referral system might be proper strategies to shorten the latency in PD diagnosis in China.

## Abbreviations

H&Y: Hoehn & Yahr
HAMA: Hamilton Anxiety Scale
HAMD: Hamilton Depression Scale
MMSE: Mini-mental State Exam
NMSquest: Non-motor symptoms questionnaire
NMSs: Non-Motor Symptoms
PD: Parkinson’s Disease
UPDRS: Unified Parkinson’s Disease Related Scale
